# Interpretable many-class decoding for MEG

**DOI:** 10.1016/j.neuroimage.2023.120396

**Published:** 2023-11-15

**Authors:** Richard Csaky, Mats W.J. van Es, Oiwi Parker Jones, Mark Woolrich

**Affiliations:** a Oxford Centre for Human Brain Activity, Department of Psychiatry, University of Oxford, OX3 7JX, Oxford, UK; b Wellcome Centre for Integrative Neuroimaging, OX3 9DU, Oxford, UK; c Department of Engineering Science, University of Oxford, OX1 3PJ, Oxford, UK; d Jesus College, OX1 3DW, Oxford, UK; e Christ Church, OX1 1DP, Oxford, UK

**Keywords:** MEG, Neuroimaging, Decoding, Machine learning, Permutation feature importance

## Abstract

Multivariate pattern analysis (MVPA) of Magnetoencephalography (MEG) and Electroencephalography (EEG) data is a valuable tool for understanding how the brain represents and discriminates between different stimuli. Identifying the spatial and temporal signatures of stimuli is typically a crucial output of these analyses. Such analyses are mainly performed using linear, pairwise, sliding window decoding models. These allow for relative ease of interpretation, e.g. by estimating a time-course of decoding accuracy, but have limited decoding performance. On the other hand, full epoch multiclass decoding models, commonly used for brain–computer interface (BCI) applications, can provide better decoding performance. However interpretation methods for such models have been designed with a low number of classes in mind. In this paper, we propose an approach that combines a multiclass, full epoch decoding model with supervised dimensionality reduction, while still being able to reveal the contributions of spatiotemporal and spectral features using permutation feature importance. Crucially, we introduce a way of doing supervised dimensionality reduction of input features within a neural network optimised for the classification task, improving performance substantially. We demonstrate the approach on 3 different many-class task-MEG datasets using image presentations. Our results demonstrate that this approach consistently achieves higher accuracy than the peak accuracy of a sliding window decoder while estimating the relevant spatiotemporal features in the MEG signal.

## Highlights

• Full-epoch decoding models achieve higher accuracy than sliding window models.
• Temporal, spatial, and spectral patterns are extracted from full-epoch models.
• A single multiclass model can be used for pairwise decoding without retraining.
• Learning the dimensionality reduction of features for decoding improves on PCA.

## 1 Introduction

Decoding external stimuli from neuroimaging data, such as Magnetoencephalography (MEG) and Electroencephalography (EEG), has gained increasing attention in recent years (Kay et al., 2008, Cichy et al., 2014). Decoding studies tend to prioritise increasing the discriminatory power (accuracy) between stimuli, e.g. in brain–computer interface (BCI) applications (Koizumi et al., 2018, Cooney et al., 2019a, Défossez et al., 2022), or gaining interpretable insights as to where and when stimuli are represented in the brain (Cichy et al., 2014, Cichy et al., 2016). These latter approaches are often referred to as multivariate pattern analysis (MVPA), and typically make use of linear, sliding-window decoders. This allows for the extraction of the interpretable spatiotemporal features that drive the decoding; for example, allowing for the estimation of a decoding accuracy time course (Cichy et al., 2014, Cichy et al., 2016, Cichy and Pantazis, 2017, Lappe et al., 2013, Higgins et al., 2022a, Higgins et al., 2022b). However, it has been demonstrated that, as one would expect, discriminatory power is also important for the effectiveness of MVPA (Guggenmos et al., 2018). Hence, there is a need in MVPA for decoding methods that improve decoding performance, while maintaining the ability to reveal the spatiotemporal features that underlie the decoding. This is especially the case for datasets with many classes.

One possibility for increasing decoding performance is to abandon the use of sliding window approaches and instead use full epoch decoding. Here, we refer to the 500 ms following stimulus presentation as the full-epoch. While it is generally good to increase the time window for decoding, as we will later show in the results, using a longer window than 500 ms might actually be detrimental. Decoding full-epoch trials has been explored most typically within the context of potential brain–computer interface (BCI) applications, for example in language tasks (Koizumi et al., 2018, Cooney et al., 2019a, Cooney et al., 2019b, Hultén et al., 2021, Dash et al., 2020a, Défossez et al., 2022) and motor tasks (Schirrmeister et al., 2017, Dash et al., 2020b, Elango et al., 2017). In contrast with the decoding employed in MVPA, BCI applications often use nonlinear multiclass models (Lawhern et al., 2018). These will generally have good discriminatory power (accuracy), but this comes at the expense of poor interpretability, and are thus not directly useful for MVPA.

Within BCI research, dimensionality reduction is often done with established supervised methods such as Common Spatial Patterns (CSP) (Blankertz et al., 2007), or Riemannian classifiers (Barachant, 2014). However, these methods do not work well when the data contains a high number of classes. Here, our main contribution is a method for doing supervised dimensionality reduction end-to-end within a neural network optimised for the classification task. We have found that the features learned by the neural network can be used to also train a standard LDA model, increasing performance substantially over either unsupervised feature reduction or the supervised Riemannian method.

Some promising approaches have been investigated recently to make full-epoch models more interpretable, such as the linear forward transform (Haufe et al., 2014). However, this approach can only be applied to linear models, and is not designed for tens or hundreds of classes. Another option is to apply full-epoch and sliding window decoding on the same data in order to get both perspectives, e.g. in Ling et al. (2019). Nonetheless, it would be hugely beneficial if a single decoding approach could be used without a loss in performance on both BCI and MVPA with a high number of conditions.

Taking together the aforementioned issues, we propose an approach that can improve decoding accuracy through the use of full-epoch multi-class decoding, while still being able to reveal the underlying spatiotemporal features that drive the decoding. This allows us to consider and investigate the use of neural network decoding models, and we also show the benefit of using supervised feature reduction. We limit our investigations to linear models, leaving nonlinear models for future work. Importantly, to allow access to interpretable features, we make use of permutation feature importance (PFI). PFI is a general technique which can be used to assess which parts of the input contribute the most to the predictions of any black-box model (Altmann et al., 2010). Chehab et al. (2022) have demonstrated the effectiveness of PFI in analysing how certain language features like word frequency affect the forecasting performance of MEG data at various temporal and spatial locations, leveraging a trained encoding model. Deep learning-specific interpretation methods have also been proposed in the context of M/EEG decoding (Schirrmeister et al., 2017, Lawhern et al., 2018)

We assess the proposed approach by systematically comparing it with sliding window decoding on three MEG datasets with visual tasks, finding that our full-epoch decoding outperforms sliding window decoding in terms of accuracy. We then compare PFI with standard alternatives and find that PFI is able to extract the same kind of dynamic temporal, spatial, and spectral information. To be clear, PFI is an established method in the literature and here we simply use it as a tool for interpretability. We do suggest some novel ways of applying PFI detailed in Section 2.5, such as in the spectral domain.

In short, the aforementioned contributions achieve the best of both worlds: a single multiclass decoding model trained on full epochs, empirically good performance, and clear interpretability from an MVPA viewpoint. This approach promises to be useful for both the BCI researcher and the neuroscientist trying to gain insight into the underlying brain activity in a particular task and external stimuli set.

## 2 Material and methods

### 2.1 Data

In this study, we used three visual MEG datasets: two similar datasets from Cichy et al. (2016) and one additional dataset from Liu et al. (2019). The datasets have been collected with appropriate consent from participants and ethical review by Cichy et al. (2016) and Liu et al. (2019), and do not contain any personal information. 15 subjects view 118 and 92 different images, respectively in the first two datasets, with 30 repetitions for each image. The third dataset is part of a larger replay study, and we only use the portion of the data where images are presented in random order for 900 ms. Here, 22 subjects view 8 different images, with 20–30 repetitions for each image (depending on the subject). The image sets used in the three datasets are different. We obtained the raw MEG data directly from the authors to run our preprocessing pipeline with MNE-Python (Gramfort et al., 2013). The 118-image and 92-image data are also available publicly in epoched form.^1^ We bandpass filtered raw data between 0.1 and 25 Hz and downsampled to 100 Hz. As recommended by prior work the sampling rate is 4 times higher than the lowpass filter (Higgins et al., 2022a). This is done so that representational alias artefacts are eliminated from the sliding window decoding time courses. We also applied whitening, which involved transforming the data with PCA to remove covariance between channels while retaining all components. The PCA was fit on the training set only but applied to both training and test sets.

Many papers have shown that visual information processing in the brain primarily operates in lower frequency ranges. Specifically, theta (4–7 Hz), alpha (8–12 Hz), and beta (13–30 Hz) bands have been implicated in various aspects of visual processing, including object recognition, visual attention, and perceptual decision-making (Klimesch, 1999, Engel and Fries, 2010, Zoefel and VanRullen, 2017). Therefore, a lowpass filter of 25 Hz captures these important frequency bands while reducing the influence of higher frequency signals that are less likely to be related to visual processing.

MEG data, like all bioelectrical signals, are often contaminated by various sources of noise. High-frequency noise, particularly above 30 Hz, often originates from sources outside the brain, such as muscle activity or environmental electromagnetic fields (Gross et al., 2013). By using a 25 Hz lowpass filter, we can significantly reduce these non-brain noise contributions, thereby improving the signal-to-noise ratio and enhancing the detectability of the brain’s visual responses.

While there are meaningful neuronal signals at frequencies above 30 Hz (e.g., gamma-band activity), decoding these high-frequency signals from MEG data can be challenging due to lower signal-to-noise ratios. Therefore, unless the specific research question involves high-frequency bands, applying a 25 Hz lowpass filter simplifies the data and focuses the analysis on the most relevant and easily interpreted signals. It also allows reducing the sampling rate, and thus the dimensionality of the data which is an important factor for achieving good classification performance with machine learning.

In the first two datasets, image presentation lasted for 500 ms with an average inter-trial interval of 0.95 s. In order to analyse the data using machine learning models, we created two versions of each dataset. The first version consisted of full epochs, with input examples having a shape of [50, 306] (or [90, 273] for the 8-image dataset), where 306 and 273 correspond to the number of MEG channels and 50 and 90 correspond to the number of time points during image presentation. The second version consisted of sliding windows, with input examples having a shape of [10, 306] (or [10, 273] for the 8-image dataset). In this case, we partitioned each trial into overlapping 100 ms time windows between 0 and 1000 ms post-stimulus and trained separate models on each time window partition as is normally done in the MVPA literature. The difference between consecutive windows was 1 timestep 10 ms. As a result, 90 independent sliding window models were trained for each dataset. In the rest of the paper we use the term “raw” to refer to the pre-processed time domain signal, as opposed to other non-time domain input features.

As opposed to some previous work using a wavelet transform of the trial as features for sliding window decoding (Higgins et al., 2022a), here we use the raw set of timepoints within the respective 100 ms window. This means that we rely more on the decoder to extract relevant frequency information rather than directly providing such information in the input. We did compare our approach with the wavelet features and found the latter to be somewhat inferior (see Inline Supplementary Figure 6). A more recent approach, termed superlets transform (Moca et al., 2021, Jorntell and Kesgin, 2023) has been shown to improve classification results by mitigating the time vs. frequency resolution problem (Bârzan et al., 2022). However, a full comparison between different time–frequency features is out of the scope of this paper, as our main comparison between sliding-window and full-epoch decoding is performed at the raw data level.

### 2.2 Neural network with supervised dimensionality reduction (NN)

**Fig. 1:**
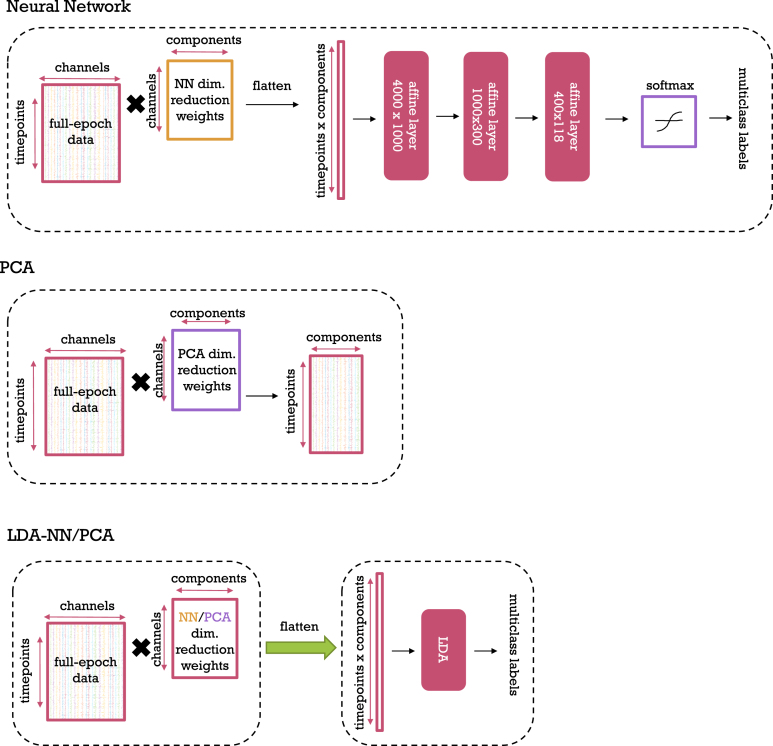
Our Neural Network, PCA, and LDA-NN/PCA methods from top to bottom. Dashed boxes represent separate processing steps, i.e. in the case of LDA-NN and LDA-PCA the respective dimensionality reduction is first used to compute the input features, which are then used to train the LDA model.

The Neural Network (NN) method is a four-layer, fully-connected linear neural network which is only run on the full-epoch dataset (Fig. 1). The first layer performed a learnable dimensionality reduction, where the full epoch data of dimensions [time points × channels] was multiplied by a weight matrix of shape [channels × components], with components (80) being less than channels. This process is similar to principal component analysis, but in this case, the dimensionality-reducing weight matrix and the decoding model are trained simultaneously; therefore, the dimensionality reduction is optimised for the classification objective. To be clear, the input size to the first layer, and thus the dimensionality of this layer, depends on the time window size and number of channels which can be different for each dataset. After the first layer, the data was flattened and three affine transformations were applied in sequence (see Fig. 1 for dimensionalities). The final layer had an output dimension equal to the number of classes, and the logits from this layer were passed through a softmax function for classification. We chose the intermediate hidden sizes (1000 and 300) to be roughly equally distanced (multiplicatively) between the input and output dimensions of the network (4000 and 118). This rationale was employed for the 118-image dataset primarily and we did not change the hidden sizes for the other two datasets.

The model was trained using cross-entropy loss (Good, 1952) for multiclass classification and included dropout between layers during training (Srivastava et al., 2014). It is worth noting that, as no nonlinearities were used, the model could be replaced with a single affine transformation during evaluation. However, deep linear neural networks are known to have nonlinear gradient descent dynamics that change with each additional layer (Saxe et al., 2013); both the learnable dimensionality-reduction layer and the use of dropout impose additional constraints on the weight matrix during learning.

### 2.3 LDA with unsupervised dimensionality reduction (LDA-PCA)

The LDA-PCA approach has two variants: one that is full-epoch, and one that uses a sliding window. In the full-epoch version, PCA is used to do unsupervised dimensionality reduction on the channel dimension of the full-epoch data as an initial, separate step (Fig. 1). The resulting PCA-reduced data matrix, which has a shape of [timepoints x components] is flattened and then used to train a multiclass classifier using LDA.

In the sliding window version, the [timepoints x components] PCA-reduced data matrix is separated into [100 ms x 80] windows. The data within each window is then flattened in the same manner as in the full-epoch version and fed into separate LDAs that are distinct to each window.

### 2.4 LDA with pre-learnt supervised dimensionality reduction (LDA-NN)

In the LDA-NN method, the PCA dimensionality-reducing weight matrix from PCA is replaced with the use of the dimensionality-reducing weight matrix extracted from the pre-trained NN approach (Fig. 1). As in LDA-PCA, this weight matrix is then applied to project the data to a [time points x components] shape, after which an LDA model is applied. In the same manner, as LDA-PCA, LDA-NN also has full-epoch and sliding window versions.

### 2.5 Permutation feature importance

To investigate the temporal dynamics of visual information processing, we utilised permutation feature importance (PFI) on our trained models. PFI is a standard tool in the literature, and our novelty lies in an empirical comparison with more established MVPA methods, and novel ways of applying it, such as in the spectral domain. Specifically, we applied PFI to a trained full-epoch LDA-NN by using sliding windows of 100 ms with 1 time point shift for each trial. The information in each window was disrupted by permuting the data across the channel dimension separately for each time window. For instance, if the window was centred around 50 ms post-stimulus, the information within that window would be disrupted from 0 to 100 ms post-stimulus compared to the original trial, while the rest of the timepoints in the trial remained unchanged. We then evaluated the trained LDA-NN on each of these disrupted trials and compared the accuracy to the original accuracy obtained with the original trials. The greater the accuracy decrease for a trial with disrupted information in a specific time window, the more crucial that time window is to the model’s performance and, therefore, the more information it contains relevant to the model’s objective of discriminating between images. By repeating this analysis for all time windows, we obtain a temporal profile of the information content, similar to the method of training separate models on individual time windows.

In terms of assessing spatial information content, we followed a similar methodology, albeit with modifications. Here, the disruption involved permuting the data across time points within each channel individually. The outcome of this operation is a sensor space map detailing the decrease in accuracy, which serves as a metric for the visual information content. This map was then compared with others generated by evaluating the per-channel accuracy of individual LDA models trained on the full epoch of each respective channel. Conceptually, this method can be seen as sliding a window (or “search-light”) across the spatial domain, similar to the previous time-based approach. In practice, we ran spatial PFI across sensors (2 gradiometers and 1 magnetometer in the same position) instead of channels, thus permuting these 3 channels together and obtaining a single metric for them. This allows for more robust results. An alternative would be to permute the gradiometers and magnetometers separately but using a spatial neighbourhood of nearby sensors for smoothing.

Additionally, we illustrated the extraction of spatiotemporal information by utilising PFI. The method involved choosing a window that spanned both space (across 4 sensors with 2 gradiometers and 1 magnetometer each, totalling 12 channels) and time (a 100 ms window) simultaneously. The spatial window contains the 2 gradiometers and 1 magnetometer on three sides of the sensor in question. The disruption of the information within this spatial–temporal window was achieved by permuting the data values across both dimensions. To cover all possible combinations, this window was then slid across all channels and time points, resulting in a spatiotemporal discriminative information content profile. This comprehensive profile allowed us to understand how the disruption of specific spatiotemporal windows impacts the performance of the trained model, therefore highlighting the importance of those windows in discriminating between visual stimuli.

Finally, we introduce *spectral PFI* to assess the effect of different frequency bands on the visual discrimination objective. First, the data in each channel of each trial is Fourier transformed, and the Fourier coefficients are permuted across channels for each frequency (or frequency band). Then, the inverse Fourier transform is computed, obtaining a trial with disrupted information in specific frequency bands. By applying this method to all frequency bands, we obtained a spectral information content profile, similar to the method of training separate LDA models on features from individual frequency bands (Higgins et al., 2022a). Similar to spatiotemporal PFI we can combine spatial and spectral PFI, by running spectral PFI on a neighbourhood of 4 sensors at a time (spatial window) to assess the spectral information content of individual MEG channels. We call this spatio-spectral PFI.

Previous work in our lab applied sliding window decoding in combination with spectral decoding (i.e., training separate models on individual frequency bands), thus assessing the temporo-spectral information content (Higgins et al., 2022a). In order to make comparisons with this work, we developed temporo-spectral PFI. Specifically, after training the full epoch decoding model, we compute the short-time Fourier transform of the entire epoch, using the same parameters as in Higgins et al. (2022a), i.e., a 100 ms Hamming window with maximal overlap. We then permuted the channel dimension of one frequency band and one window at a time, leaving the other frequency bands and windows unchanged. Finally, we perform the inverse short-term Fourier transform on the full epoch to get the time domain data back (i.e., channels-by-timesteps), on which the trained decoding model is then applied. By repeating this over all frequency bands and time windows we can obtain the temporo-spectral PFI profile.

### 2.6 Experimental details

The primary evaluation metric for the three datasets is classification accuracy across the respective number of classes (118, 92, or 8). The main focus of our analysis was on the 118 and 92-image datasets, with the 8-image dataset, included to demonstrate the effects of a much smaller sample size. All of the main results using our decoding methods (NN, LDA-NN, LDA-PCA) are multiclass. For all analyses, separate models were fit to separate subjects. Training and validation splits were created in a 4:1 ratio for each subject and class, with classes balanced across the splits. The NN approach was trained for 2000 epochs (full passes of the training data as opposed to epochs in the sense of MEG trials) using the Adam optimiser (Kingma and Ba, 2015). The high number of epochs was selected as this allowed the training accuracy to converge to almost 100%, while the validation accuracy also converged to a stable value for most participants. The dimensionality reduction layer and PCA were both set to 80 components, as it is slightly higher than the inherent dimensionality reduction of MaxFilter which is applied to the MEG data, and thus contains more than 99% of variance. We briefly tried our pipeline with 60 components as well on 1 subject and found similar results. The output layer’s dimensionality was equal to the number of classes in the corresponding dataset. Dropout was set to 0.7 and applied before each of the three hidden layers.

Validation data was not used for early stopping, and the trained NN dimensionality reduction weight matrix (used in LDA-NN) was extracted after the full 2000 epochs of training on the training data. For the LDA models, the shrinkage parameter was set to “auto” using the sklearn package. Comparisons of interest over methods were evaluated using Wilcoxon signed rank tests, with within-subject pairing and subject-level mean accuracies over validation examples as the samples. We used Bonferroni correction to correct for multiple comparisons. The PyTorch package was used for training (Paszke et al., 2019), and several other packages were utilised for analysis and visualisation (Pedregosa et al., 2011, Virtanen et al., 2020, Harris et al., 2020, McKinney, 2010, Waskom, 2021, Hunter, 2007). Code written for our analysis can be accessed at https://github.com/ricsinaruto/MEG-transfer-decoding.

## 3 Results

### 3.1 Full-epoch models achieve better accuracy than sliding-window decoding

**Fig. 2:**
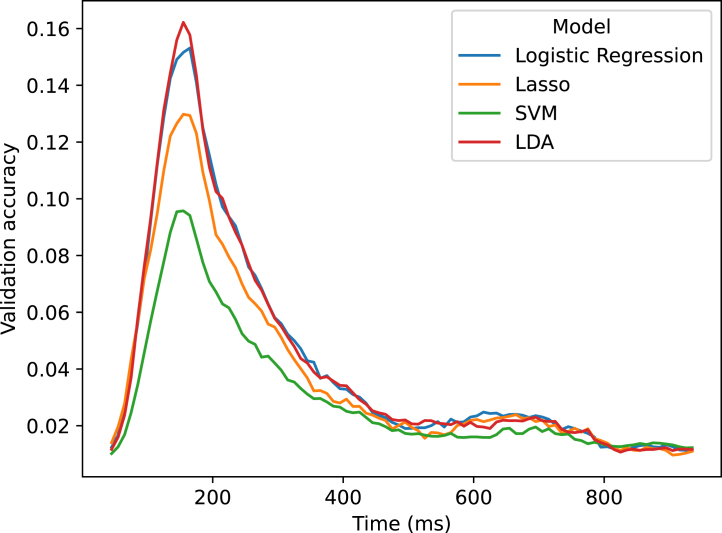
Comparing different sliding window models trained on PCA features on the 118-image dataset for multiclass decoding. The sliding window size is 100 ms. Results are averaged across subjects.

We set out to test whether full-epoch decoding is better than timepoint-by-timepoint and sliding-window decoding, which are common practices in the MEG literature (Carlson et al., 2011, Carlson et al., 2013, Su et al., 2012, Ramkumar et al., 2013, Cichy et al., 2017, Grootswagers et al., 2017, Kurth-Nelson et al., 2016, Liu et al., 2019, Higgins et al., 2022a). We wanted to make sure that our classifier of choice, LDA is at least as good as other commonly used models for multiclass decoding, including support vector machines (SVM), linear discriminant analysis (LDA), logistic regression, and Lasso. The results, depicted in Fig. 2, indicate that LDA and logistic regression exhibited comparable performance (no statistical difference) and performed better than the other 2 examined models. For this reason, and as described in the methods, we used LDA in all further analyses for comparing different classification strategies.

**Fig. 3:**
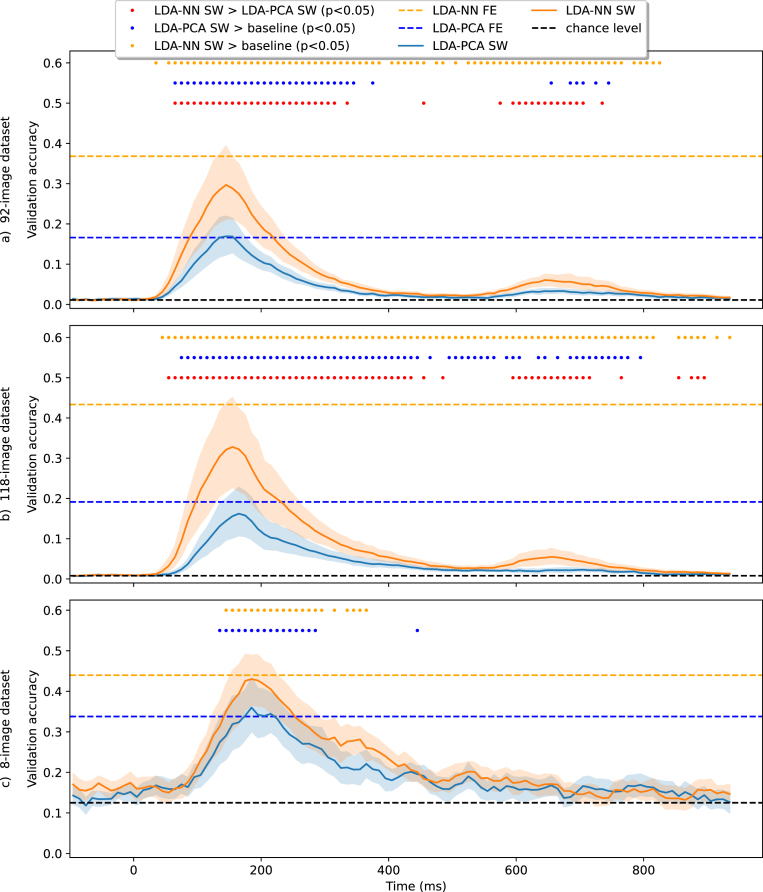
Models trained on the sliding-window versions of the 92-class dataset (top), 118-class dataset (middle) and 8-class dataset (bottom) for multiclass decoding. Wilcoxon signed-rank tests are reported between sliding window LDA-NN and LDA-PCA. We also ran Wilcoxon signed-rank tests between the first timepoint of LDA-NN and LDA-PCA and all other timepoints. This shows statistical significance compared to a baseline level. FE stands for full-epoch models, and SW stands for sliding window models. The blue and orange dotted lines are placed at the average performance of full-epoch LDA-NN and LDA-PCA, respectively. All statistical tests are Bonferroni corrected for multiple comparisons across all time points (i.e. p-values are multiplied by 90). Shading indicates the 95% confidence interval across subjects. For the full-epoch results, please see Fig. 4 for distributions across subjects. LDA-NN is better across almost all time points than LDA-PCA, and full-epoch accuracy is higher than peak sliding window accuracy for both LDA-NN and LDA-PCA (except in the 92-class and 8-class datasets).

The performance of multiclass full-epoch models was compared to that of sliding-window decoding for both LDA-PCA and LDA-NN on the three datasets in Fig. 3. The peak performance of sliding-window decoding was observed at 150–160 ms post-stimulus for the 92 and 118-image datasets, and at 200 ms post-stimulus for the 8-image dataset. These findings are broadly consistent with previous research on the temporal dynamics of visual information processing in MEG (Cichy et al., 2014, Cichy et al., 2016, Cichy and Pantazis, 2017, Higgins et al., 2022a, Liu et al., 2019, Guggenmos et al., 2018). For the 92 and 118-image datasets a second smaller peak was observed around 650–660 ms post-stimulus. As the image presentation is switched off at exactly 500 ms, we reason that the second peak is due to the brain reacting to this event. The first peak is observed 150–160 ms post-stimulus onset, while the second peak occurs 150–160 ms post-stimulus offset.

Across subjects, the full-epoch LDA-PCA approach demonstrated significantly higher accuracy than the best sliding-window LDA-PCA approach on the 118-class dataset (3.1% increase, p < 1e-4). On the 92-class dataset, no significant difference was observed between these models, though full-epoch LDA-PCA still outperformed the sliding-window version at most time windows. A similar comparison between full-epoch LDA-NN and peak sliding-window performance showed that full-epoch models had higher accuracy on both the 92- and 118-class datasets (7.1% and 10.5% increase, respectively, p < 1e-4). The tests were corrected for multiple comparisons across time points. These results indicate that training a model on the full epoch generally leads to better performance than using the best sliding-window model, except for the LDA-PCA approach on the 92-image dataset. However, as noted in the following section, it is advisable to use an LDA-NN model in any case.

Our results could be affected by the choice of window size for the sliding window LDA (100 ms). Thus, we repeated the sliding window LDA for different window sizes, including a window of 1 sample (i.e., timepoint-by-timepoint decoding), and the results are presented in Inline Supplementary Figure 2. We found that as the window size increased accuracy improved reaching full-epoch performance with a 200 ms window but the accuracy profile became more distorted and the peak shifted compared to the results obtained with a single time point.

Finally, on the 8-image dataset, the full-epoch model had higher accuracy than the peak sliding-window model, though this difference was not significant. It should be noted that the reduced effectiveness of the full-epoch model on this dataset may be due to both the longer epoch of 900 ms and the smaller amount of data. This can lead to overfitting due to a larger number of features and fewer examples.

### 3.2 Supervised dimensionality reduction is better than PCA

**Fig. 4:**
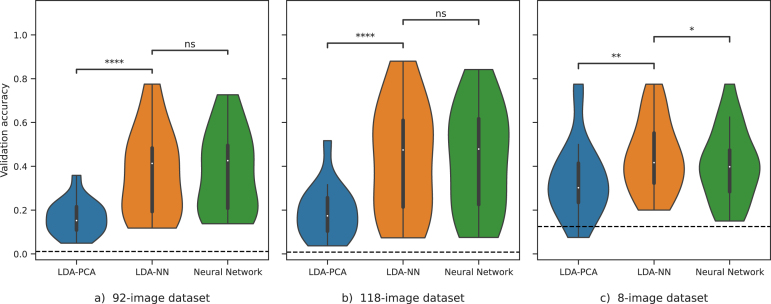
Models trained on the full-epoch versions of the 92-class (left), 118-class (middle), and 8-class (right) datasets for multiclass decoding. The violin plot distributions are shown over the mean individual subject performances. The dashed black line represents the chance level. Wilcoxon signed-rank tests are shown where 4 stars mean p < 1e-4, and 3 stars mean p < 1e-3. “ns” means that the p-value is higher than 0.05.

We next investigated the effect of incorporating a learned, supervised dimensionality reduction layer in our models, i.e. a dimensionality reduction optimised to aid a downstream classification task. We, therefore, modified the LDA-PCA approach by replacing the unsupervised dimensionality reduction performed by PCA with the supervised dimensionality reduction (of equal dimensionality) from the Neural Network (NN) approach, as described in Section 2. We refer to this modified approach as LDA-NN. As shown in Fig. 4, this simple change resulted in a significant improvement in performance (20.2% for the 92-class dataset and 24.2% for the 118-class dataset, p < 1e-4). We also assessed the performance of the pure NN model and found that it has a similar performance to LDA-NN. In other words, the supervised dimensionality reduction effectively eliminated the performance gap between the LDA and the Neural Network (NN) approach.

The sliding window versions of LDA-PCA and LDA-NN are also compared in Fig. 3. Across most time points (and all time points around the 2 peaks), LDA-NN is significantly better than LDA-PCA, when Bonferroni corrected for multiple comparisons across time points. Similar conclusions can be drawn on the 8-image dataset, although LDA-NN is better than the NN approach, possibly due to the reduced performance of neural networks on small datasets in general. In summary, our results suggest that using a full-epoch LDA-NN or a simple linear Neural Network results in the best performance across all datasets and that the feature reduction should be learned in a supervised manner for both the LDA and Neural Network models.

### 3.3 Full-epoch models contain the same kind of temporal and spatial information as sliding window decoding

**Fig. 5:**
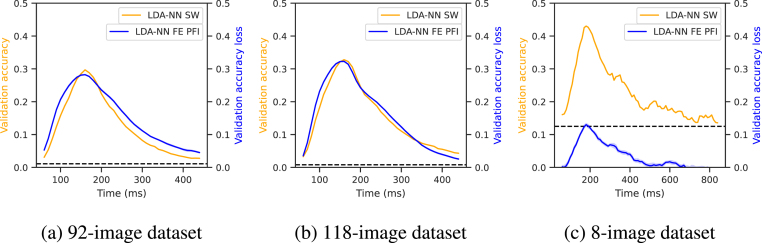
Comparison of multiclass sliding window LDA-NN (orange) and the temporal PFI of multiclass full-epoch LDA-NN (blue) across the three datasets. Results are averaged across all subjects in the respective datasets, and shading indicates 95% confidence interval across permutations for PFI. Chance level for LDA-NN SW is indicated with a dashed line.

One of the benefits of sliding window or time-point-by-time-point decoding is that it is straightforward to obtain a time course of decoding accuracy (e.g., Fig. 3), allowing for interpretation of the temporal dynamics of neural representations. Here we show that full epoch decoding in combination with permutation feature importance (PFI) can give the same qualitative information. The results presented in Fig. 5 indicate that temporal PFI applied to a full-epoch LDA-NN model produces temporal profiles similar to those obtained using sliding window LDA-NN models with a window size of 100 ms across all three datasets. The peak sliding window performance also aligns well with the peak accuracy loss for PFI.

**Fig. 6:**
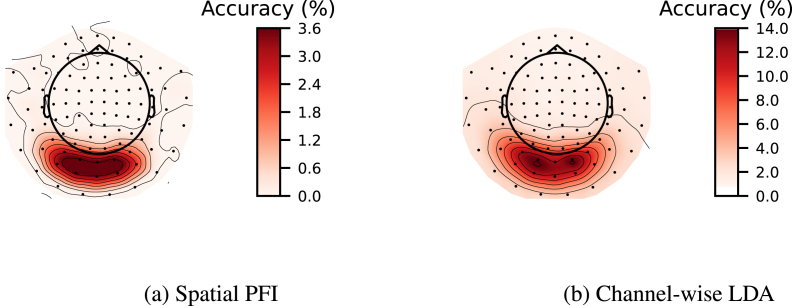
Comparison of multiclass channel-wise LDA model (b) with the spatial PFI of multiclass full-epoch LDA-NN (a). Spatial maps are averaged across all 15 subjects on the 118-image dataset. Both PFI and the channel-wise LDA model are run on 3-channels in the same location at a time (1 magnetometer and 2 gradiometers).

In addition, we investigated the ability of PFI to accurately capture spatial information by applying it to a full-epoch LDA-NN model on the 118-image dataset. To do this, we permuted time points from the gradiometers and magnetometers located at the same position in the MEG data simultaneously to obtain a single sensor space map. We compared these to the maps obtained by training separate LDA models on the full epoch of the same three sensors (2 gradiometers and 1 magnetometer). This approach can be viewed as a sliding window across space. All PFI results are averaged over the accuracy losses of individual subjects, which can somewhat smear both spatial and temporal profiles. The results, shown in Fig. 6, demonstrate good alignment between the accuracy loss of spatial PFI and per-sensor accuracy of LDA-NN, indicating that PFI can effectively recover spatial information content.

**Fig. 7:**
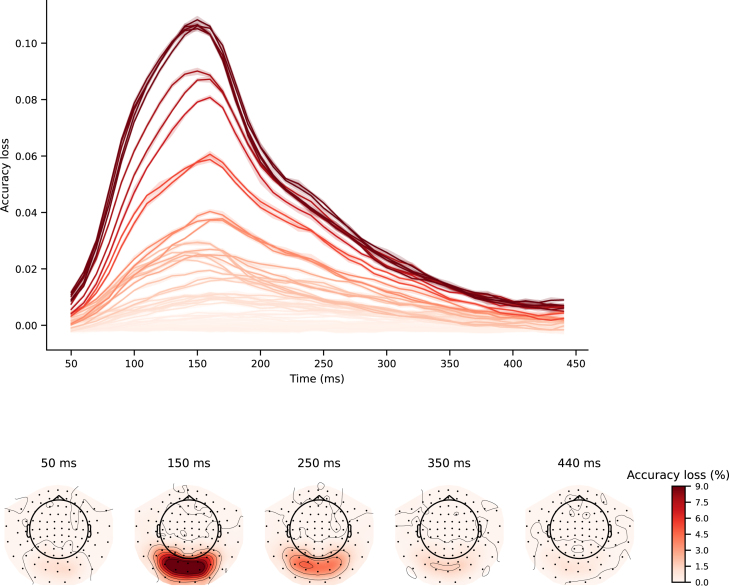
Spatiotemporal PFI of multiclass full-epoch LDA-NN on the 118-image dataset. Blocks of 4-channel neighbourhoods and 100 ms time windows are shuffled to obtain a spatial and temporal profile jointly. Each line in the temporal profile corresponds to a sensor, and each sensor space map is obtained with a time window centred around the respective time point. The colour map of the upper plot is based on the colouring of sensors at 150 ms in the lower plot. The shading in the upper plot is across the 10 permutations used for PFI and indicates the 95% confidence interval. Both temporal and spatial profiles are averaged over subjects.

We also employed PFI to extract spatiotemporal information jointly from a trained full-epoch LDA-NN model on the 118-image dataset. Specifically, we used a 100 ms time window and a 4-channel spatial window (i.e., the 2 gradiometers and 1 magnetometer on three sides of the sensors in question) for each time point and channel, shuffling the values within these blocks. This allowed us to unravel the temporal and spatial information simultaneously, showing that only channels located in the visual area exhibited the characteristic temporal profile and that there was a gradient with channels further from the visual area displaying progressively lower peak accuracy loss (Fig. 7). Additionally, we observed that the temporal evolution of the sensor space maps showed the visual area sensors to be consistently the most important for the decoding objective across all time points. A full animation of the temporal evolution of the sensor space maps is provided in Inline Supplementary Video 1. In theory, the sliding window LDA and the per-channel LDA approach could be combined to get a similar spatiotemporal profile, where each LDA model is trained on the sliding window of 4 channels at a time. However, in practice accuracy might suffer substantially with so few input features, and it would be computationally taxing considering the amount of LDA models required to train. Overall, PFI proved to be a useful technique for investigating full-epoch data and obtaining spatiotemporal information similar to what can be obtained from individual sliding window models.

**Fig. 8:**
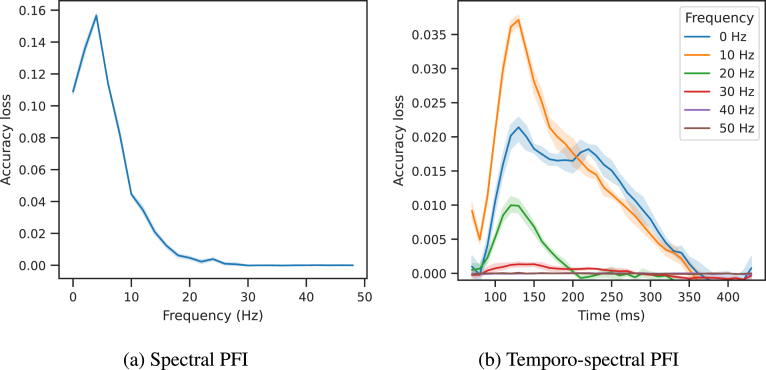
Spectral PFI (left) and temporospectral PFI (right) of multiclass full-epoch LDA-NN on the 118-image dataset. Shading indicates 95% confidence interval across permutations. Results are averaged across subjects.

Finally, Fig. 8(a) presents our spectral PFI results averaged over subjects. This shows a clear peak of spectral information content at 4 Hz, after which the power rapidly declines with increasing frequency. However, it should be noted that, because of the sampling rate of the data and the size of the epochs, the frequency resolution is only 2 Hz. This means that the apparent 4 Hz peak is due to the 1 Hz highpass used for preprocessing the data, and so in actuality there is simply a 1/f characteristic, as is expected in MEG data. We have confirmed this by plotting the psd of the raw (bandpassed) data with a matched frequency resolution, and found the same peak at 4 Hz, which shows that this is an artefact of the frequency resolution.

We present temporospectral PFI in Fig. 8(b), which reveals temporal information content within individual frequency bands, in an alternative manner to using separate LDA models trained on wavelet features (Higgins et al.). All frequency values represent bands centred around the respective frequencies, except the 0 Hz band which represents the true 0 Hz signal, i.e. the average over the time window. For computing the STFT we followed the same setup as in Higgins et al. (2022a). Because we are using a 100 ms window (10 timesteps) for computing the STFT the frequency resolution is 10 Hz. When permuting a specific time window, we also permuted the frequency content of the time window right before and after, to obtain a smoother temporal profile.

As expected from the standard temporal PFI, the temporal peak is between 100 and 150 ms. Spectrally, higher frequency bands tend to be less and less useful to the decoding objective, confirming the observations of Higgins et al. (2022a). However, we think both the figure in Higgins et al. (2022a), and the temporospectral PFI analysis are slightly misleading, as they could be interpreted as having a peak in information content in the 10 Hz band. As observed in Fig. 8(a) this effect is explained simply by the 1/f characteristic. Because of the poor frequency resolution, both lower and higher frequencies are represented in the 10 Hz band, thus all it shows is the 1/f characteristic, and the reason why it is higher than the “0 Hz” band is because the 0 Hz band contains solely the true 0 Hz content. A potentially better approach to disentangling time–frequency information content would be to bandpass the data first into specific frequency bands, then train our decoding model and compute the temporal PFI on each bandpassed data version.

We note that it is expected that we would find little to no signal above 25 Hz, because of the lowpass filter we have employed. In later timepoints (> 200 ms) the 0 Hz band seems to be slightly more important than the 10 Hz band, potentially meaning that the classifier relies more on average rather than oscillatory activity after the visual peak. Similar to spatiotemporal PFI we can combine spatial and spectral PFI to assess the spectral information content of individual MEG channels (see Inline Supplementary Figure 5).

## 4 Discussion

We made the following contributions in this work. We showed empirically that full-epoch models achieve higher accuracy than sliding window decoding models. We showed that temporal, spatial, and spectral brain activity patterns related to stimulus discrimination can be extracted for any black-box many-class full-epoch model. The main novelties of this paper are the supervised dimensionality reduction of input features, within a neural network optimised for the classification task, and the benefits of many-class full-epoch models. We have shown that this improves performance substantially. Next, we discuss each result in more detail.

We have found that training a single full-epoch model for multiclass decoding is effective in improving decoding performance, and have shown how this can be used while still providing neuroscientific insights by using PFI to learn which features are contributing to the decoding accuracy. Our results show that a full-epoch model generally performs better than individual sliding window models for visual decoding tasks, and the magnitude of this effect increases with the size of the dataset. The time-efficiency benefits of using a full-epoch model are significant, as training sliding window models takes roughly 10 times longer than a single full-epoch model for a 100 ms time window with a 100 Hz sampling rate. Additionally, our analysis of different window sizes (see Supplementary Material) showed that while larger window sizes may improve performance, they are not effective in accurately capturing the temporal profile of information content. It has also been suggested that using equal-length time windows for all trials does not account for trial-by-trial variability, and Vidaurre et al. (2018) proposed time-resolved decoding using a Hidden Markov Model to segment trials along the time dimension. This approach still involves training multiple models on multiple time windows. We, therefore, recommend using full-epoch models, as they only need to be trained once and contain information from all potentially useful time windows. After training any desired window size can be selected for temporal or spatial investigations through PFI, providing good decoding performance and dynamic spatiotemporal resolution without the need for retraining.

We also found that incorporating a supervised dimensionality reduction layer is essential for good decoding performance when using linear neural networks and LDA models. This can be used as a drop-in replacement over standard unsupervised dimensionality reduction typically done with PCA. Within BCI research, dimensionality reduction is often done with established supervised methods such as Common Spatial Patterns (CSP) (Blankertz et al., 2007). Supervised variants of PCA have also been introduced, but not for MEG data (Kobak et al., 2016). A gold standard approach to designing BCI decoders is the use of a Riemannian classifier that also performs a supervised class separation (Barachant, 2014). Importantly, these methods rely on a separate feature extraction step before applying the classifier, whereas we wanted to include both steps in a single neural network to allow end-to-end training. Furthermore the Riemannian classifier with Xdawn only works well when the number of classes is low, thus not applicable to the datasets in this paper.

We compared PFI results from a full-epoch model with those from individual models trained on either separate time or spatial windows. This demonstrated that PFI can effectively extract both temporal and spatial information, and can also be used to investigate the interaction between these two dimensions. We also introduced a new technique whereby PFI can be used to extract spectral discriminatory information content and confirmed that this matches previous work training individual models on separate frequency bands. PFI is a particularly flexible technique, as it can be applied to nonlinear models and temporal or spatial resolution can be chosen post-hoc without the need for retraining. The performance of full-epoch nonlinear decoding and corresponding PFI analysis will be explored in future work. PFI can also be applied to individual conditions or single trials by rerunning with different permutations, enabling the investigation of various neuroscientific questions. Other methods for obtaining temporal and spatial information from trained models, such as the Haufe transform, are limited to linear models and do not provide trial-level patterns (Haufe et al., 2014). As opposed to the statistical nature of PFI, the Haufe transform directly maps the weights of a linear decoding model to input patterns, thus showing which parts of the input are the most important for the decoding objective. However, the effectiveness of the Haufe transform in the case of tens or hundreds of classes is limited. One downside of PFI compared to the Haufe transform is that the absence of influence on the output does not necessarily mean that those parts of the input (channels or time windows) do not contain information about the target.

Using multiclass models when there is a large number of classes is useful for RSA. Computation time is also reduced compared to pairwise models, as these reuse the data for training while a multiclass model uses it only once. A method for obtaining pairwise accuracies from a multiclass model is presented in the Supplementary Material.

To conclude, when the number of classes is high, we recommend using a full-epoch multiclass model equipped with a supervised dimensionality reduction in order to achieve the best possible decoding performance while also allowing for flexibility in conducting neuroscientific investigations post-hoc such as MVPA or RSA. Our methods and recommendations scale well with data size and can be readily applied to deep learning models as well, thus bringing the applications of decoding to brain–computer interfaces and representational brain dynamics under a joint approach.

## CRediT authorship contribution statement

**Richard Csaky:** Conceptualisation, Methodology, Software, Investigation, Writing – original draft, Writing – review & editing, Visualization. **Mats W.J. van Es:** Conceptualisation, Writing – original draft, Writing – review & editing, Supervision. **Oiwi Parker Jones:** Conceptualisation, Writing – original draft, Writing – review & editing, Supervision. **Mark Woolrich:** Conceptualisation, Writing – original draft, Writing – review & editing, Supervision, Project administration.

## Declaration of competing interest

None

## Data Availability

In this study, we used three previously published visual MEG datasets: two similar datasets from Cichy et al. (2016) and one additional dataset from Ling et al. (2019). We obtained the raw MEG data directly from the authors, however the (Cichy et al., 2016) datasets are also publicly available in epoched form: http://userpage.fu-berlin.de/rmcichy/fusion_project_page/main.html.

## Appendix A Supplementary data

MMC S1 The supplementary material includes pairwise decoding results, and further sliding window and PFI comparisons.

MMC S2

## Footnotes

1 http://userpage.fu-berlin.de/rmcichy/fusion_project_page/main.html.
