# Supplementary figures and images for: Interpretable many-class decoding for MEG
Source: Neuroimage. 2023 Nov 15;282:120396. doi: 10.1016/j.neuroimage.2023.120396 (PMC10938061; doi:10.1016/j.neuroimage.2023.120396)

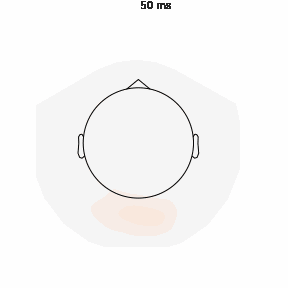

Supplement: MMC S1 — The supplementary material includes pairwise decoding results, and further sliding window and PFI comparisons. [file mmc1.zip › Inline_Supplementary_Video_1.gif]
