# Supplementary material for: Interpretable many-class decoding for MEG
Source: Neuroimage. 2023 Nov 15;282:120396. doi: 10.1016/j.neuroimage.2023.120396 (PMC10938061; doi:10.1016/j.neuroimage.2023.120396)
Supplement: MMC S2 [file mmc2.pdf]

1 Supplementary Material

1.1 Multiclass decoding models evaluated for pairwise decoding are on par with direct pairwise models

We have demonstrated that multiclass full-epoch models are better than sliding window models while maintaining the same level of spatiotemporal information. Here we wish to highlight an additional advantage of using multiclass full-epoch models. Researchers frequently use pairwise models to analyse the representational differences between individual conditions or groups of conditions, such as in representational similarity analysis (RSA). However, this approach can be computationally intensive, especially when dealing with a large number of classes.

Here, we show how we can utilise a single trained multiclass full-epoch LDA-NN model to predict pairwise accuracy scores. This is done by iteratively taking all pairs of conditions, computing the predicted probabilities across all classes for each trial, and selecting the condition with the higher probability (of the two conditions) as the predicted class. By comparing this to the ground-truth labels, we can obtain pairwise accuracy scores for each pair of conditions. In Inline Supplementary Figure 1, we compared the results of this method with those obtained by training individual pairwise (full epoch LDA-NN) models as is typical in the literature. For the 92 and 118-image datasets, the multiclass model achieved slightly, but significantly higher pairwise accuracy than the individual pairwise models. The difference was not significant for the 8-image datasets. Therefore, using a multiclass model can yield pairwise results that are similar to or even better than those obtained from individual pairwise models. This provides a much more efficient way of obtaining pairwise accuracies for the purposes of RSA.

1.2 Window size comparison

In Inline Supplementary Figure 2, we examined the impact of the sliding window size on the results of our LDA-NN models. We trained models using sliding window sizes of 10ms, 100ms, 200ms, 300ms, and 400ms. As expected, using a single time point (10ms) resulted in lower accuracy compared to a 100ms window. As the window size increased, we observed two trends. First, accuracy improved and the peak accuracy of a 200ms window already reached the full-epoch level. Second, the accuracy profile became more distorted and the peak shifted compared to the results obtained with a single time point. In some cases, full-epoch performance was even exceeded by a few percentage points with a 300ms window. This may not be surprising, as a larger window that focuses on the most significant part of the input results in fewer features compared to using the full epoch. However, it is advisable to avoid using a window larger than 100ms in sliding window analysis due to its

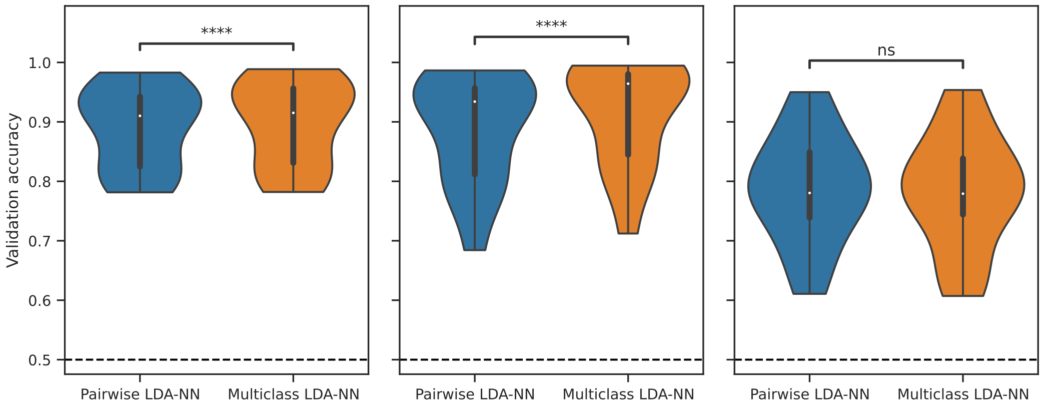

a) 92-image dataset b) 118-image dataset c) 8-image dataset

Inline Supplementary Figure 1: Comparison of pairwise full-epoch LDA-NN models (blue) with multiclass models evaluated for pairwise classification (orange) across the three datasets. In all datasets except the 8-image dataset, multiclass models evaluated in a pairwise fashion are significantly better (****, p<1e-4). The violin plot distributions are shown over the mean individual subject performance. The dashed line represents chance level.

distortion and lower temporal resolution. One potential solution could be to combine the sliding window models with PFI analysis, but this would be inefficient. We can therefore conclude that using a full-epoch model is the optimal solution, even if it results in slightly lower accuracy. Additionally, we expect that with larger datasets, full-epoch models would outperform sliding window models regardless of window size, as the ratio of features to examples would be reduced.

1.3 Inverse temporal and spatial PFI

We investigated an alternative method of performing PFI, referred to as inverse PFI. This method is not common in the literature, but could be interesting from an MVPA viewpoint. Inverse PFI differs from standard PFI in that it shuffles values outside a specified time window, rather than within it. Standard PFI assesses the impact of disrupting information within a specific window on performance and therefore reveals the importance of that window for discriminating between images. In contrast, inverse PFI investigates performance when all information outside a specified window is disrupted, thereby providing insight into the performance that can be achieved using only the information contained within the time window. The temporal PFI results for both standard and inverse PFI are presented in Inline Supplementary Figure 3. While both approaches are similar to the standard sliding window LDA profile, there are some differences as well. We also conducted this analysis in the spatial domain, the results of which are shown in Inline Supplementary Figure 4. In this domain, the inverse PFI approach exhibits less contrast between visual channels and other channels

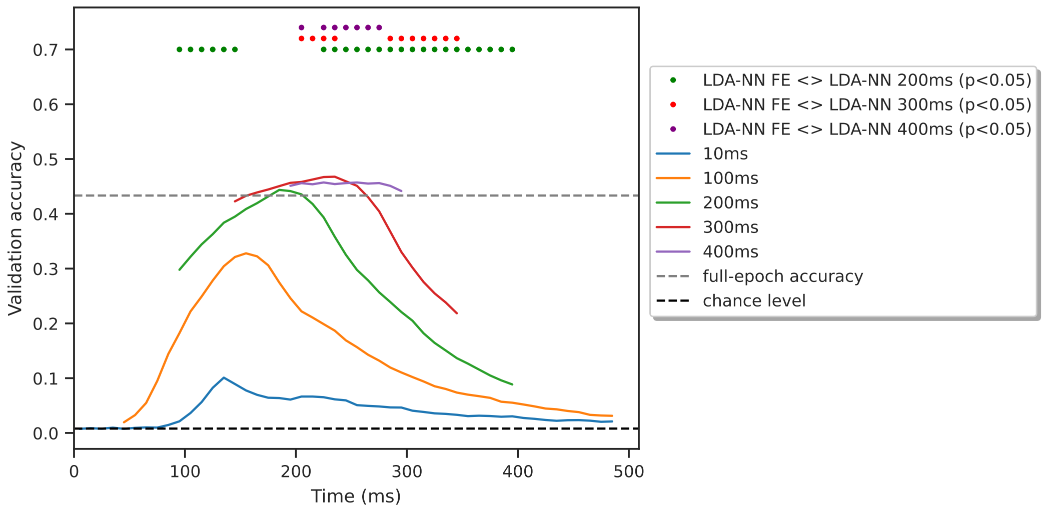

Inline Supplementary Figure 2: Comparing sliding window LDA-NN with different window sizes on the 118-image dataset. Results are averaged across subjects. Wilcoxon signed-rank tests are reported between the sliding window models and the full-epoch model, Bonferroni corrected for all comparisons in the figure.

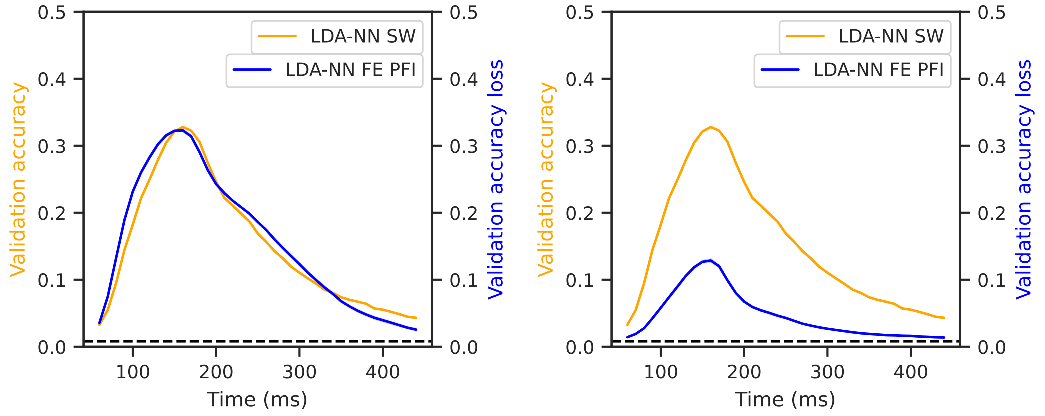

(a) Standard PFI

(b) Inverse PFI

Inline Supplementary Figure 3: Comparison of multiclass sliding window LDA-NN (orange) with standard temporal PFI (a) and inversed temporal PFI (b) using a trained LDA-NN model on the 118-image dataset. Results are averaged across subjects, and shading indicates the 95% confidence interval across permutations for PFI. Chance level is indicated by the dashed line.

but appears to distinguish between visual channels more similarly to channel-wise LDA than standard PFI. It is not the aim of this study to determine which approach is superior, as both seem to have their merits.

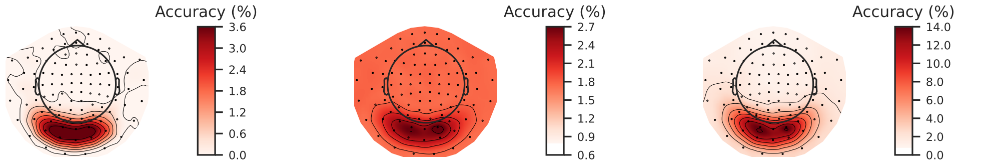

(a) Standard PFI (b) Inverse PFI (c) Channel-wise LDA

Inline Supplementary Figure 4: Comparison of channel-wise LDA model (c) with the standard spatial PFI (a) and inverse spatial PFI (b) of full-epoch multiclass LDA-NN. Results are averaged across all 15 subjects on the 118-image dataset. Both PFI and the channel-wise LDA model are run on 3-channels in the same location at a time (1 magnetometer and 2 gradiometers).

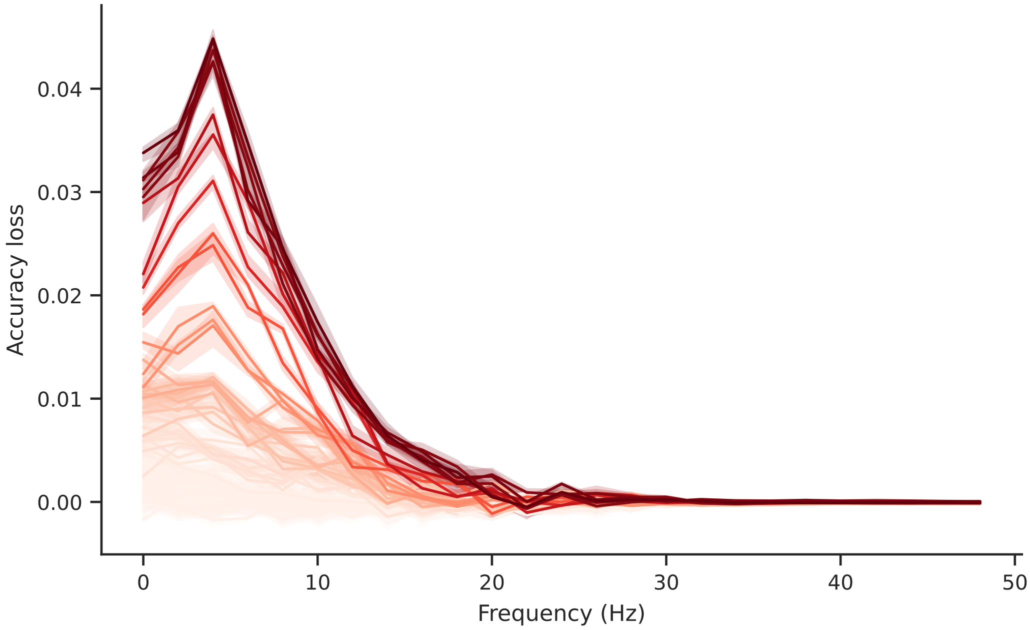

Inline Supplementary Figure 5: Spatiospectral PFI of multiclass full-epoch LDA-NN on the 118-image dataset, averaged over subjects. Blocks of 4-channel neighbourhoods are shuffled in each frequency to obtain the per-channel frequency profile. Each line corresponds to a sensor. The color map of the upper plot is based on the overall spatial PFI of each sensor, i.e. sensors with high spatial PFI accuracy loss are shown as darker red. The shading is across the permutations used for PFI and indicates the 95% confidence interval.

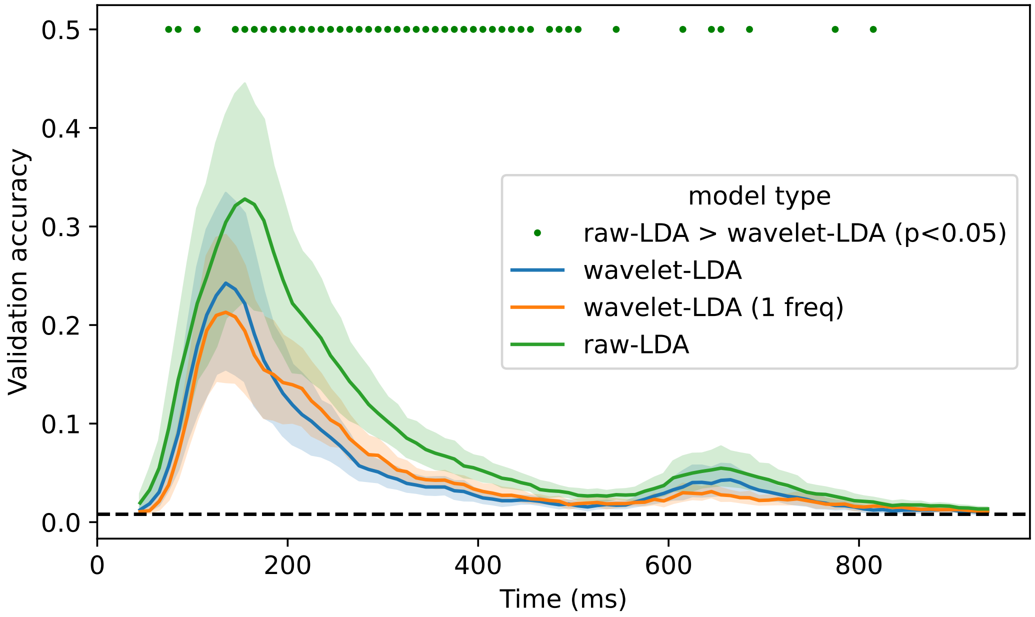

Inline Supplementary Figure 6: Comparison of our sliding window LDA-NN approach with LDA-NN using wavelet features on the 118-image dataset. The wavelet features are computed after the dimensionality reduction. A hamming window of 10 timesteps was used with an overlap of 9 timesteps. wavelet-LDA corresponds to using a concatenation of all frequency bands for training the LDA model, and wavelet-LDA (1 freq) uses a single frequency band (10Hz). We selected this band based on previous results in Higgins et al. (2022), achieving the best decoding performance using this band only. Results are averaged across subjects, and shading indicates the 95% confidence interval across subjects.
